# Distinguishing fibromyalgia from rheumatoid arthritis and systemic lupus in clinical questionnaires: an analysis of the revised Fibromyalgia Impact Questionnaire (FIQR) and its variant, the Symptom Impact Questionnaire (SIQR), along with pain locations

**DOI:** 10.1186/ar3311

**Published:** 2011-04-08

**Authors:** Ronald Friend, Robert M Bennett

**Affiliations:** 1 Fibromyalgia Research Unit, Oregon Health & Science University, 3455 SW Veterans Road, Portland, OR 97239, USA; 2 Department of Psychology, 100 Nicholls Road, Stony Brook University, Stony Brook, NY 11794-2500, USA

## Abstract

**Introduction:**

The purpose of this study was to explore a data set of patients with fibromyalgia (FM), rheumatoid arthritis (RA) and systemic lupus erythematosus (SLE) who completed the Revised Fibromyalgia Impact Questionnaire (FIQR) and its variant, the Symptom Impact Questionnaire (SIQR), for discriminating features that could be used to differentiate FM from RA and SLE in clinical surveys.

**Methods:**

The frequency and means of comparing FM, RA and SLE patients on all pain sites and SIQR variables were calculated. Multiple regression analysis was then conducted to identify the significant pain sites and SIQR predictors of group membership. Thereafter stepwise multiple regression analysis was performed to identify the order of variables in predicting their maximal statistical contribution to group membership. Partial correlations assessed their unique contribution, and, last, two-group discriminant analysis provided a classification table.

**Results:**

The data set contained information on the SIQR and also pain locations in 202 FM, 31 RA and 20 SLE patients. As the SIQR and pain locations did not differ much between the RA and SLE patients, they were grouped together (RA/SLE) to provide a more robust analysis. The combination of eight SIQR items and seven pain sites correctly classified 99% of FM and 90% of RA/SLE patients in a two-group discriminant analysis. The largest reported SIQR differences (FM minus RA/SLE) were seen for the parameters "tenderness to touch," "difficulty cleaning floors" and "discomfort on sitting for 45 minutes." Combining the SIQR and pain locations in a stepwise multiple regression analysis revealed that the seven most important predictors of group membership were mid-lower back pain (29%; 79% vs. 16%), tenderness to touch (11.5%; 6.86 vs. 3.02), neck pain (6.8%; 91% vs. 39%), hand pain (5%; 64% vs. 77%), arm pain (3%; 69% vs. 18%), outer lower back pain (1.7%; 80% vs. 22%) and sitting for 45 minutes (1.4%; 5.56 vs. 1.49).

**Conclusions:**

A combination of two SIQR questions ("tenderness to touch" and "difficulty sitting for 45 minutes") plus pain in the lower back, neck, hands and arms may be useful in the construction of clinical questionnaires designed for patients with musculoskeletal pain. This combination provided the correct diagnosis in 97% of patients, with only 7 of 253 patients misclassified.

## Introduction

Rheumatoid arthritis (RA), systemic lupus erythematosus (SLE) and fibromyalgia (FM) are usually easily discriminated on clinical examination, but have several overlapping features that make their differentiation more problematic in epidemiological surveys. For instance, pain, fatigue and morning stiffness are commonly reported in all three disorders. The current study was stimulated by the increasing interest in developing questionnaires that can accurately predict the occurrence of FM in both epidemiological and clinical settings [1-5]. During the evaluation of an updated version of the Fibromyalgia Impact Questionnaire (FIQR), we compared its properties in patients with FM with those in patients with RA, SLE and major depressive disorder (MDD) [6]. Although the primary intent of this analysis was to validate the FIQR as a useful instrument in assessing the overall impact and severity of FM, it was incidentally noted that it had some diagnostic utility in differentiating FM from SLE and RA [6]. A slightly modified version of the FIQR, the Symptom Impact Questionnaire (SIQR), was used for the SLE and RA groups. The SIQR is identical to the FIQR, but does not contain any reference to FM [6]. For instance, the total SIQR score discriminated FM from these three disorders, with FM having a total FIQR score of 56.6, whereas RA had a score of 27.9, SLE had a score of 29.5 and MDD had a score of 17.3. We also reported on pain in 24 locations in the FIQR study to confirm that FM patients who had not been seen recently still had widespread pain. While this pain location questionnaire was not used in FIQR scoring, the number of pain locations was, as expected, much higher in FM patients: 16 pain sites for patients with FM compared to 6 sites in patients with RA, 7 sites in patients with SLE, 4 sites in patients with MDD and 1.6 sites in healthy controls. The objective of the current study was to identify individual SIQR symptoms and pain locations that best discriminated FM patients from RA/SLE patients in this data set. Doing so provides some pointers as to which pain sites and common symptoms may best discriminate FM from RA/SLE in patient questionnaires.

## Materials and methods

The data analyzed are taken from the revision of the FIQ (the FIQR) and its non-FM variant, the SIQR. The Bennett *et al. *[6] study compared a sample of healthy controls with FM, RA, SLE and MDD patients. All data were analyzed using STATISTICA version 8 software (StatSoft, Inc. Tulsa, OK, USA). In the present study, we compared the data from 202 FM patients, 20 SLE patients and 31 RA patients. The MDD group was not used, because the sample size of 11 was too small for classification purposes.

The SIQR questionnaire is provided in Table 1. The SIQR differs from the original FIQ [7] in that it has modified function questions and new items related to memory, tenderness, balance and environmental sensitivity. It consists of three domains: Function (nine items), Overall Impact (two items) and Symptoms (ten items) that are scored on a scale from 0 to 10, with 10 being the most severe (Table 1). The 24 pain locations that were used to confirm that FM patients still had widespread pain were as follows: left shoulder, right shoulder, left jaw, right jaw, left upper back, right upper back, left arm, right arm, left hand, right hand, left lower back, right lower back, left hip, right hip, left thigh, right thigh, left knee, right knee, left foot, right foot, mid-upper back, mid-lower back and front of chest and neck (see Table 2). These locations were designed to reflect a distribution of widespread pain in terms of 10 axial pain locations above and below the waist (neck, left and right jaw, left and right upper back, left and right lower back, mid-upper back, mid-lower back and chest), 8 proximal limb locations (shoulders, arms, hips and thighs) and 6 distal limb locations (hands, feet and knees).

**Table 1:** The Symptom Impact Questionnaire (SIQR)

Domain 1: For each question, place an "X" in the box that best indicates how much difficulty you have experienced in doing the following activities during the past 7 days. If you did not perform a particular activity in the last 7 days, rate the difficulty for the last time you performed the activity. If you can't perform an activity, check the last box.			
Brush or comb your hair	No difficulty	□ □ □ □ □ □ □ □ □ □ □	Very difficult
Walk continuously for 20 minutes	No difficulty	□ □ □ □ □ □ □ □ □ □ □	Very difficult
Prepare a homemade meal	No difficulty	□ □ □ □ □ □ □ □ □ □ □	Very difficult
Vacuum, scrub or sweep floors	No difficulty	□ □ □ □ □ □ □ □ □ □ □	Very difficult
Lift and carry a bag full of groceries	No difficulty	□ □ □ □ □ □ □ □ □ □ □	Very difficult
Climb one flight of stairs	No difficulty	□ □ □ □ □ □ □ □ □ □ □	Very difficult
Change bed sheets	No difficulty	□ □ □ □ □ □ □ □ □ □ □	Very difficult
Sit in a chair for 45 minutes	No difficulty	□ □ □ □ □ □ □ □ □ □ □	Very difficult
Go shopping for groceries	No difficulty	□ □ □ □ □ □ □ □ □ □ □	Very difficult
**Domain 2: For each of the following 2 questions, check the one box that best describes the overall impact of any medical problems over the last 7 days**.			
My medical problems prevented me from accomplishing goals.	Never	□ □ □ □ □ □ □ □ □ □ □	Always
I was completely overwhelmed by my medical problems	Never	□ □ □ □ □ □ □ □ □ □ □	Always
**Domain 3: For each of the following 10 questions, check the one box that best indicates the intensity of the following common symptoms over the last 7 days**.			
Please rate your level of pain	No pain	□ □ □ □ □ □ □ □ □ □ □	Unbearable pain
Please rate your level of energy	Lots of energy	□ □ □ □ □ □ □ □ □ □ □	No energy
Please rate your level of stiffness	No stiffness	□ □ □ □ □ □ □ □ □ □ □	Severe stiffness
Please rate the quality of your sleep	Awoke rested	□ □ □ □ □ □ □ □ □ □ □	Awoke very tired
Please rate your level of depression	No depression	□ □ □ □ □ □ □ □ □ □ □	Very depressed
Please rate your level of memory problems	Good memory	□ □ □ □ □ □ □ □ □ □ □	Very poor memory
Please rate your level of anxiety	Not anxious	□ □ □ □ □ □ □ □ □ □ □	Very anxious
Please rate your level of tenderness to touch	No tenderness	□ □ □ □ □ □ □ □ □ □ □	Very tender
Please rate your level of balance problems	No imbalance	□ □ □ □ □ □ □ □ □ □ □	Severe imbalance
Please rate your level of sensitivity to loud noises, bright lights, odors and cold	No sensitivity	□ □ □ □ □ □ □ □ □ □ □	Extreme sensitivity

Scoring: (1) Sum the scores for each of the three domains (Function, Overall and Symptoms). (2) Divide domain 1 score by 3, divide domain 2 score by 1 (that is, unchanged) and divide domain score 3 by 2. (3) Add the three resulting domain scores to obtain the total SIQR score (range, 0 to 100).

**Table 2:** Percentage pain site response for RA, SLE and FM with the calculated differences between groups (including the combined RA/SLE group)

Location	Healthy (*n *= 204)	FM (*n *= 202)	RA (*n *= 31)	SLE (*n *= 20)	RA minus SLE	RA/SLE (*n *= 51)	FM minus RA/SLE
Shoulders	14%	76%	32%	25%	7%	29%	48%
Jaws	4%	36%	3%	10%	-7%	7%	30%
Arms	6%	69%	23%	10%	13%	16%	53%
Hands	5%	64%	81%	73%	9%	77%	-13%
Hips	11%	79%	29%	28%	2%	28%	51%
Thighs	4%	55%	0%	0%	0%	0%	55%
Knees	10%	64%	39%	53%	-14%	46%	18%
Feet	12%	50%	46%	63%	-17%	54%	-4%
Lateral upper back	6%	82%	15%	23%	-8%	19%	64%
Lateral lower back	8%	80%	23%	20%	3%	22%	59%
Mid upper back	4%	77%	13%	15%	-2%	14%	63%
Mid lower back	16%	79%	10%	25%	-15%	18%	62%
Front of chest	4%	54%	10%	15%	-5%	13%	42%
Neck	16%	91%	29%	55%	-26%	42%	49%
Peripheral	7%	55%	28%	29%	-1%	28%	26%
Axial	9%	77%	17%	25%	-9%	21%	56%

**Note: **Minus scores in the RA minus SLE column indicate that the SLE group had higher scores on that item. Minus scores in the FM minus RA/SLE column indicate that the RA/SLE group had higher scores on that item. FM, fibromyalgia; RA, rheumatoid arthritis; SLE, systemic lupus erythematosus.

### Patients

The data from this study were derived from the same patients who had completed the FIQR and SIQR questionnaires for the previously published paper [6]. Ethical approval for reanalysis of these data was not required by our institutional guidelines. All participants had completed online informed consent forms, and the study was conducted in accordance with the Declaration of Helsinki.

### Statistical analyses

First, the frequency and means comparing FM, RA and SLE participants on all pain sites and SIQR variables are presented and analyzed. Second, multiple regression analysis was conducted to identify the significant pain site and SIQR predictors of group membership (FM and RA/SLE). A two-step analytic and variable reduction procedure was used. Standard multiple regression analysis identified the significant and unique predictors of group membership, thereby reducing the number variables from 35 to 15. Then stepwise multiple regression analysis was performed, which ordered these 15 variables according to their maximal statistical contribution in predicting FM and RA/SLE membership. Partial correlations assessed their unique contribution, and two-group discriminant analysis provided a classification table [8].

## Results

### Pain site frequency

The 10 left- and 10 right-side pain locations (for both right and left sides: jaws, shoulders, upper outer back, lower outer back, arms, hands, hips, thighs and feet) were highly correlated (range, *rs *= 0.66 to 0.85; mean, *r *= 0.77). To avoid multicollinearity and reduce the number of variables, the left and right sides were averaged to form 10 variables, which, together with the 4 axial sites (mid-upper back, mid-lower back, neck and front of chest), formed the 14 pain sites used as predictors. Table 2 shows the percentages of healthy controls and FM, RA, SLE and RA patients, as well as RA combined with SLE patients (RA/SLE), who reported pain at these 14 pain sites. The data for healthy patients are also included to provide a baseline for comparison. The first four of columns Table 2 show the pain site percentages in healthy controls and FM, RA and SLE patients. To discern whether there was much difference between RA and SLE patients, the fifth column shows the calculated difference between these two groups. The sixth column shows the combined RA and SLE figures (RA/SLE), and the last column shows the FM minus RA/SLE difference, a measure of discriminatory sites. Interestingly, there was not a very large discordance between pain sites in RA and SLE patients, except for neck pain, which was endorsed by 55% of SLE patients versus 29% of RA patients (*P *< 0.0001). As might be expected, hand pain was more common in RA patients, but foot and knee pain were unexpectedly more common in SLE patients. FM patients generally reported many more pain locations than RA/SLE patients, except, as might be expected, for the hands and feet. FM patients frequently reported pain in the extremities and thus a report of hand and/or foot pain does not necessarily discriminate FM from RA/SLE patients. The bottom two rows show the average percentage of patients with pain in peripheral and axial locations. FM patients more often reported axial pain, with an average frequency of 77% in axial locations compared to an average frequency of 21% among RA/SLE patients (*P *< 0.0004). Interestingly, peripheral pain locations were more prevalent in FM patients than in RA/SLE patients (55% vs. 28%, *P *< 0.0002). A notable pain location was the thigh; this was never reported in RA/SLE patients, whereas 55% of FM patients had pain in this region. Jaw pain was reported in 36% of FM patients but in only 7% of RA/SLE patients (*P *< 0.0001). It is relevant to note that the FM minus RA/SLE differences are really "zero order relations" and do not necessarily identify unique differences after controlling for other predictors (see section, 'Forward stepwise regression analysis of pain sites and SIQR predictors of group membership').

The fairly close concordance of pain sites in RA and SLE patients provides some justification for merging them into a single group (RA/SLE) to increase statistical power and permit regression and discriminant analyses.

### SIQR item frequency

Table 3 shows the SIQR scores of healthy controls and FM, SLE and RA patients, as well as RA patients combined with SLE patients (RA/SLE). The computed total SIQR score (bottom row) and the function, overall and symptom averages were also computed. As in the case of the pain site frequency table, the last column (FM minus RA/SLE) provides some indication of the possible items that are most discriminatory between FM and RA/SLE. The highest differences (≥3.5) were seen for difficulty cleaning floors, discomfort on sitting for 45 minutes and tenderness to touch, all of which were more severe in FM patients. The averaged total SIQR score in FM patients was 56.6 versus 28.6 in RA/SLE patients (*P *< 0.0001). The RA minus SLE column shows very little difference between RA and SLE patients (all < 0.8), with the exceptions of environmental sensitivity (-2.9, 1.6 vs. 4.5; *P *< 0.001), which was more of a problem for the SLE group, and climbing one flight of stairs (1.3, 3.6 vs. 2.3; *P *= 0.06), which was more difficult for the RA group. Overall, these results, along with the pain site frequency findings, provide reasonable justification for merging the RA and SLE groups in the following analyses.

**Table 3:** Individual SIQR questions for RA, SLE and FM with the calculated differences between RA and SLE and between FM and the combined RA/SLE groups

SIQR question	Healthy (*n *= 204)	FM (*n *= 202)	RA (*n *= 31)	SLE (*n *= 20)	RA minus SLE	RA/SLE (*n *= 51)	FM minus RA/SLE
Brush or comb hair	0.1	2.4	0.9	0.8	0.1	0.8	1.6
Walk continuously for 20 minutes	0.6	5.7	3.4	2.2	1.2	2.9	2.8
Prepare a homemade meal	0.2	4.3	1.2	1.4	-0.2	1.3	3.0
Vacuum, scrub or sweep floors	0.6	6.5	2.8	2.5	0.3	2.7	3.8
Lift and carry a bag full of groceries	0.4	5.6	2.6	3.3	-0.7	2.9	2.7
Climb one flight of stairs	0.5	5.6	3.6	2.3	1.3	3.1	2.5
Change bed sheets	0.4	5.5	2.4	2.2	0.2	2.3	3.2
Sit in a chair for 45 minutes	0.7	5.6	1.5	1.6	-0.1	1.5	4.1
Go shopping for groceries	0.4	5.6	2.5	2.4	0.1	2.4	3.2
**Function (average)**	0.4	5.2	2.3	2.1	0.2	2.2	3.0
Achieve goals	0.7	5.7	2.7	3.1	-0.4	2.8	2.9
Feel overwhelmed	0.7	5.2	2.5	3.3	-0.8	2.8	2.4
**Overall (average)**	0.7	5.5	2.6	3.2	-0.6	2.8	2.7
Pain	1.5	6.0	3.9	4.1	-0.2	3.9	2.1
Energy	2.6	6.8	5.1	5.1	0.0	5.1	1.7
Stiffness	2.1	6.7	4.5	4.1	0.4	4.4	2.3
Sleep	3.8	7.6	5.4	5.5	-0.1	5.5	2.1
Depression	1.7	4.6	1.8	1.8	0.0	1.8	2.8
Memory	1.7	5.9	2.7	3.4	-0.7	3.0	2.9
Anxiety	1.8	4.5	1.9	2.6	-0.7	2.2	2.3
Tenderness	1.0	6.9	3.4	2.5	0.9	3.0	3.9
Balance	0.7	4.8	2.0	1.8	0.2	1.9	2.9
Sensitivity	1.5	6.2	1.6	4.5	-2.9	2.8	3.4
**Symptoms (average)**	1.8	6.0	3.2	3.5	-0.3	3.3	2.7
**Total SIQR score**	12.4	56.6	27.9	29.6	-1.7	28.6	28.0

**Note: **Minus scores in the RA minus SLE column indicate that SLE group had higher scores on that item. Higher scores indicate more impairment or higher level of symptoms. FM, fibromyalgia; RA, rheumatoid arthritis; SLE, systemic lupus erythematosus.

### Pain site and SIQR predictors of FM and RA/SLE group membership and classification analyses

A preliminary standard multiple regression analysis was performed with the 14 pain site variables and 21 SIQR variables to identify which variables were uniquely and statistically associated with FM vs. RA/SLE group membership. This analysis identified 11 significant variables: neck, *P *< 0.0009; arms, *P *< 0.002; hands, *P *< 0.003; lower back, *P *< 0.046; thigh, *P *< 0.033; feet, *P *< 0.007; tenderness to touch, *P *< 0.0001; cleaning floors, *P *< 0.002; sitting for 45 minutes, *P *< 0.003; depression, *P *< 0.01; and anxiety, *P *< 0.034. Four other variables, mid-lower back pain (*P *< 0.08), feeling overwhelmed (*P *< 0.065), poor memory (*P *< 0.09) and environmental sensitivity (*P *< 0.09), were marginally significant and were retained in the final regression analysis model so as not to preclude their possible contribution in a final analysis. The seven pain site and eight SIQR variables were then entered into a forward stepwise regression analysis (Table 4) to identify which variables best discriminated the FM from the RA/SLE group. Table 5 shows their unique contribution (partial correlations) when the other 14 variables were controlled for. Last, discriminant function analysis was used to classify FM and RA/SLE individuals according to this final variable list (Table 6).

**Table 4:** Stepwise multiple regression showing 15 predictors ranked in order of magnitude in predicting group membership (FM or RA/SLE)

Predictors	Step and number of variables included	Multiple R	Multiple R^2^	R^2 ^change	*P *value for predictor variable
Mid-lower back	1	0.540	0.291	0.291	0.00000
Tenderness to touch	2	0.637	0.406	0.115	0.00000
Neck	3	0.689	0.474	0.068	0.00000
Arms	4	0.712	0.507	0.033	0.00007
Hands	5	0.747	0.558	0.051	0.00000
Lateral lower back	6	0.758	0.575	0.017	0.00168
Sitting for 45 minutes	7	0.768	0.589	0.014	0.00367
Feeling overwhelmed	8	0.775	0.601	0.012	0.00750
Depression	9	0.784	0.615	0.014	0.00365
Sensitivity	10	0.791	0.626	0.011	0.00855
Thighs	11	0.797	0.635	0.009	0.01471
Feet	12	0.804	0.647	0.012	0.00529
Cleaning floors	13	0.806	0.649	0.003	0.16326
Anxiety	14	0.807	0.652	0.002	0.19893
Memory	15	0.809	0.654	0.002	0.21899

**Note: **This forward stepwise regression analysis used 15 predictors which combined to produce a multiple R = 0.809 (last row, column 2). This accounted for 65% of variance associated with group membership (column 3). FM, fibromyalgia; RA/SLE, combined rheumatoid arthritis and systemic lupus erythematosus group.

**Table 5:** Forward stepwise multiple regression analysis showing zero order (Pearson's *r*) and partial correlations

Predictors	Pearson's *r*	Partial *r*	*P *value (partial *r*)
Mid-lower back	-0.540	-0.129	0.0458
Tenderness to touch	-0.518	-0.242	0.0002
Neck	-0.518	-0.275	0.0000
Arms	-0.447	-0.261	0.0000
Hands	0.162	0.237	0.0002
Lateral lower back	-0.524	-0.191	0.0030
Sitting for 45 minutes	-0.475	-0.177	0.0060
Feeling overwhelmed	-0.314	0.274	0.0000
Depression	-0.378	-0.190	0.0031
Sensitivity	-0.422	-0.144	0.0258
Thighs	-0.474	-0.166	0.0101
Feet	0.021	0.176	0.0064
Cleaning floors	-0.452	-0.085	0.1914
Anxiety	-0.292	0.099	0.1277
Memory	-0.428	-0.080	0.2190

**Note**: Minus correlations indicate that FM patients have higher scores on predictor variable. All Pearson's correlations are significant (*N *= 253; *P *< 0.001) except hands (*P *< 0.01) and feet (*P *< 0.74).

**Table 6:** Correct classification as predicted by discriminant analysis using seven pain sites and eight SIQR variables

Group	FM	RA/SLE	Percent correct
FM (*n *= 202)	200	2	99.01%
RA/SLE (*n *= 51)	5	46	90.20%

**Note**: The combined correct classification for FM and RA/SLE = 97.23%. FM, fibromyalgia; RA/SLE, combined rheumatoid arthritis and systemic lupus erythematosus group.

### Forward stepwise regression analysis of pain sites and SIQR predictors of group membership

A forward stepwise regression model (Table 4) with 15 predictors combined to produce a multiple *r *= 0.809 (see Table 4, bottom row, column 2), accounting for 65% of variance associated with group membership (see Table 4, column 3). Additional hierarchical regression analyses (not shown) indicated that this 65% variance could be further decomposed into 30% of variance shared between SIQR and pain sites, 24% unique to pain sites and 11% unique to SIQR variables. With regard to the 15 predictors, the first 7 predictors particularly (mid-lower back pain, neck pain, arm pain, hand pain, outer lower back pain, tenderness to touch and sitting for 45 minutes) accounted for almost 60% of this variance. These seven most important predictors of group membership in order of magnitude (variance accounted for and FM vs. RA/SLE differences indicated) were mid-lower back pain (29%; 79% vs. 18%), tenderness to touch (11.5%; 6.86 vs. 3.02), neck pain (6.8%; 91% vs. 42%), hand pain (5%; 64% vs. 77%), arm pain (3%; 69% vs. 16%), outer lower back pain (1.7%; 80% vs. 22%) and sitting for 45 minutes (1.4%; 5.56 vs. 1.49). Mid- and lower-back pain, though they showed strong zero order correlation and quite different percentages in Table 2, have smaller partial correlations in Table 5 because of their shared variance as indicated by their quite strong correlation with each other (*r *= 0.56). In fact, while mid-lower back pain was the first variable to be entered into the stepwise regression analysis, being responsible for 29.1% of variance (Table 3, column 4), the corresponding partial coefficient, indicating unique contribution, was only -0.129 (Table 5, column 3). On the other hand, tenderness to touch and neck pain contributed both substantial and unique variance. It is of note that hand and foot pain, which are not much different in Table 2 and have low zero order correlations in Table 5 (-0.162 and -0.021, respectively), had stronger unique and statistically significant partial relations (0.237 and 0.176, respectively), thus indicating stronger associations with RA/SLE. It is also relevant to note that the magnitude of the FM minus RA/SLE pain site differences in Table 2 and correlations in Table 5 (which are zero order relations) are not completely reflected by the results of the multivariate regression analysis, as exemplified by the partial correlations in Table 5. Of the 14 pains sites listed in Table 3, the 5 most important pain sites in Table 5 that discriminate FM from RA/SLE are the mid- and outer lower back, neck, arms and hands. Similarly, of the 23 SIQR items, the important variables are "tenderness to touch" and "sitting in a chair for 45 minutes." While the SIQR "tenderness" variable was a strong predictor of group assignment, the SIQR "pain" variable did not distinguish FM from RA/SLE. Overall, these variables suggest that the relationship between predictors and group membership can be best described by a number of specific pain locations plus a high level of tenderness to touch.

#### Other unique predictors and considerations: pain, tenderness, and pain sites in FM and RA/SLE

Given that SIQR tenderness was an important discriminator of RA/SLE groups and SIQR pain was not, further analyses were conducted to provide some insight as to how pain, tenderness and pain sites function in relation to each other and also to FM and RA/SLE.

#### Mean differences in SIQR tenderness and SIQR pain in FM and RA/SLE

**Figure 1:**
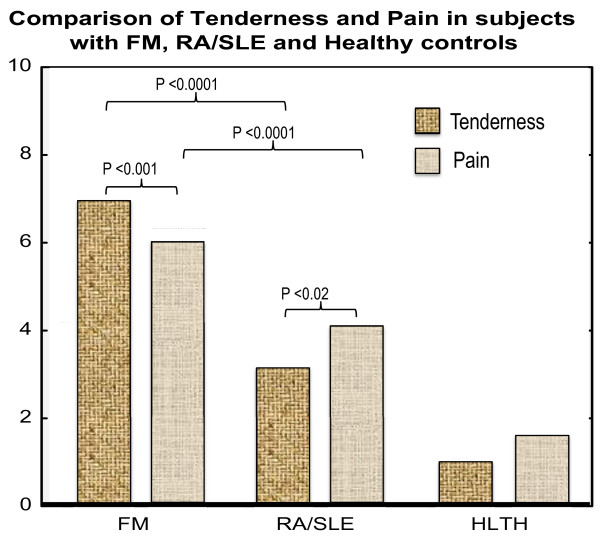
**The main effect shows that both tenderness and pain are significantly greater in fibromyalgia than in rheumatoid arthritis/systemic lupus erythematosus**. However, the interaction shows that **(a) **this difference is greater in fibromyalgia (FM) than in rheumatoid arthritis/systemic lupus erythematosus (RA/SLE) and **(b) **tenderness is more severe than pain in FM, whereas pain predominates over tenderness in RA/SLE. The healthy control values are provided for background comparison. HLTH, healthy controls.

A repeated measures 2 × 2 analysis of variance (FM, RA/SLE × tenderness, pain) was performed on the means for FM and RA/SLE. A main effect [F(1, 251) = 84.87; *P *< 0.0001)] showed that FM patients, compared with RA/SLE patients, reported significantly more tenderness (6.86 vs. 3.02; *P *< 0.001) and pain (6.01 vs. 3.94; *P *< 0.008). An interaction [F(1, 251) = 20.17, *P *< 0.0001)] comparing the two patient groups shows that this approximates a four-point difference for tenderness relative to a two-point difference for pain. These differences may in part account for why tenderness but not pain was a stronger predictor in classifying patients in the discriminant analysis. Additionally, the FM group reported more tenderness than pain (6.86 vs. 6.01; *P *< 0.001), while RA/SLE patients reported slightly more pain than tenderness (3.94 vs. 3.02; *P *= 0.019). Thus "tenderness" was rated higher by FM patients, while pain was rated higher by RA/SLE patients (see Figure 1). A χ^2 ^test indicated that 58% vs. 25% of FM and RA/SLE patients, respectively, indicated a greater tenderness than pain score (*P *< 0.001).

#### SIQR pain and SIQR tenderness prediction of total pain site

A second analysis using standard multiple regression was conducted to determine how tenderness and pain, uniquely and together, predicted total pain site scores in the FM and RA/SLE groups separately. In FM patients, pain (β = 0.277, *P *= 0.0002) and tenderness (β = 0.181, *P *= 0.013) were both independent predictors of total pain site scores (*R *= 0.389, *P *= 0.001). In the RA/SLE group, only pain (β = 0.472, *P *= 0.003) but not tenderness (β = 0.042, *P *= 0.78) predicted total pain sites (*R *= 0.497, *P *= 0.001). This demonstrates that while SIQR pain predicts pain sites in both groups, tenderness to touch predicts pain sites only in the FM group.

Along with the regression analyses, the latter analyses point to several conclusions. First, FM patients reported higher tenderness than pain scores, whereas the reverse was true of RA/SLE patients, who reported higher pain than tenderness scores. Second, tenderness to touch seems to be an important "between group" variable in discriminating FM from RA/SLE patients, whereas pain is not. Third, both pain and tenderness are independent predictors of pain sites in FM patients, whereas only pain is a predictor of pain sites in RA/SLE patients. Collectively, these analyses show that tenderness to touch plays a unique role in differentiating FM from RA/SLE and is a unique predictor of pain sites in FM patients but not in RA/SLE patients. With regard to RA/SLE patients, pain was rated higher than tenderness to touch and was correlated with pain sites, whereas tenderness was not. These findings indicate that variables predicting between-group identification do so in a different way than they do in predicting within-group severity differences. Notably, tenderness to touch plays a unique role in both differentiating FM from RA/SLE patients and in predicting FM severity (in addition to pain) among FM patients.

## Discussion

This analysis of FIQR/SIQR items and 24 pain locations provides some potentially useful pointers to questions that could be used in the construction of epidemiological questionnaires in surveys of musculoskeletal pain. The questions in the SIQR reflect the domains (pain, tenderness, fatigue, multidimensional function and sleep) that the Outcome Measures in Rheumatology Clinical Trials (OMERACT) [9] has recommended as core dimensions to be assessed in all FM clinical trials. The SIQR includes domains that are also deemed to be important by OMERACT (that is, fatigue, dyscognition, stiffness, depression and anxiety). The SIQR items relating to balance and environmental sensitivity have not been evaluated in the OMERACT process, but are some of the commonest complaints of FM patients [10].

While the classification criteria for RA, SLE and FM all require a physical examination, epidemiological surveys seldom provide for patient examination, thus the development of discriminatory questionnaires is problematical. The one physical examination criterion for FM, as per the 1990 American College of Rheumatology (ACR) classification criteria, is the finding of ≥11 of 18 designated tender points [11]. Reporting on tenderness of joints is part of the ACR and Disease Activity Score (DAS) system in the evaluation of RA severity [12,13]. One might logically surmise that the symptom of tenderness to touch that is "whole body," as in FM, would be more severe than focal joint tenderness in RA, which is what we found in this analysis. Although the finding of inflammatory arthritis in two or more joints is one of the eleven criteria used in SLE classification [14], tenderness *per se *is not part of these criteria. Thus it was of interest to note that in this analysis, tenderness to touch in SLE patients was similarly rated in RA and SLE patients (2.9 vs. 3.4).

Overall the combination of seven pain sites and eight SIQR items together produced a multiple R of 0.81 (65% variance), accounting for substantial variance in group membership, with a correct classification rate of 97%. From a conceptual perspective, it is interesting to note that the largest component of this variance (30%) was shared by pain sites and SIQR items, indicating that pain locations and SIQR dimensions are intimately connected in differentiating FM from RA/SLE. The additional unique contribution of pain sites (24%) and SIQR items (11%), particularly tenderness to touch, suggest that epidemiological surveys should consider both of these items to maximize their effectiveness. But neither pain sites nor SIQR variables alone seem sufficient to differentiate patient groups. The role of SIQR pain was different and also significant when examining within-group correlations rather than correlations across groups (pooled across groups) as described above. Both SIQR pain and SIQR tenderness to touch significantly predicted pain site scores in the FM group, while only SIQR pain predicted total pain site scores in the RA/SLE group. Furthermore, the means for SIQR tenderness to touch and SIQR pain were different, thus showing discriminant validity between FM and RA/SLE.

A notable finding in this study was that the SIQR question on tenderness to touch, along with neck pain, arm pain and hand pain, were important symptoms to consider when developing questionnaires to distinguish FM from RA or SLE. In all analyses, tenderness contributed equally with other specific pain sites to the classification of FM and RA/SLE patients. The SIQR pain variable did not help to distinguish FM from RA or SLE patients, possibly because the pain site captures pain ratings, thus making the SIQR "pain" variable redundant. This notion is supported by the observation that tenderness was correlated with pain (0.55), but was more strongly associated with group diagnosis than pain (0.52 vs. 0.35).

Nevertheless, while pain and tenderness uniquely predicted pain sites, they did not account for much variance in pain site. A more refined measure of pain locations, such as a pain VAS, one that specified the nature or quality of the pain in greater detail or one which included axial, distal and proximal subscale scores, might provide more useful information than a simple count of presence or absence of pain.

We are not aware of other survey questionnaires that ask about "tenderness to touch." However, the recent preliminary diagnostic FM criteria paper did find that a widespread pain index and muscle tenderness were the most important variables in the classification of cases and noncases of FM, although tenderness was not used in the final formulation of the criteria [4]. It seems possible that the "tenderness to touch" variable may be a useful surrogate for a tender point evaluation in musculoskeletal pain surveys without a physical examination. It is also worthy of comment that "tenderness to touch" was associated with a diagnosis of FM even when psychological variables such as depression, anxiety and "feeling overwhelmed" were controlled for in multivariate regression analyses, thus challenging the still common notion that tenderness in FM can be explained in terms of a psychiatric condition or a psychosomatic reaction. Looking backward to the 1990 ACR study, the finding of "tenderness to touch" is redolent of the "skin-fold tenderness" test, which provided odds ratios of 8.8 and 6.5, respectively, for the diagnosis of primary FM and secondary FM over controls [11].

Although FM patients had higher pain scores than RA/SLE patients (6.0 vs. 3.9), pain was not a useful between-group discriminator. We surmised that this was due to pain locations being a better discriminator. The SIQR only asks about pain in the general sense, and maybe more specific questions would be useful in epidemiological surveys. For instance, Perrot *et al. *[3] reported on the development of a rapid screening tool for FM and found that positivity for at least five of six questions ("I have pain all over my body," "My pain is accompanied by continuous and very unpleasant general fatigue," "My pain feels like burns, electric shocks or cramps," My pain is accompanied by other unusual sensations throughout my body, such as pins and needles, tingling or numbness," "My pain is accompanied by other health problems such as digestive problems, urinary problems, headaches or restless legs," and "My pain has a significant impact on my life, particularly on my sleep and my ability to concentrate, making me feel slower generally") had a sensitivity of 90.5% and a specificity of 85.7% in differentiating FM from a composite group comprising non-FM with RA, ankylosing spondylitis and osteoarthritis.

There are several limitations of the present study. The number of RA/SLE patients was small compared to the FM population (51 vs. 202). The pain locations were designed to reflect a composite of widespread pain and peripheral pain. In this respect, it may have been useful to include the wrists and ankles, joints that are commonly involved in RA. The RA and SLE patients were specifically screened for not having concomitant FM, and thus this study does not provide any useful information on that common combination, which is now known to skew the results of questionnaires such as the DAS [15]. The patients in this study were not screened for hand osteoarthritis, a condition that is found in about 80% of older adult patients [16]; however, hand pain was the only pain location that was more prevalent in RA/SLE than in FM.

While researching background information for this paper, it became apparent that very little information has been published regarding musculoskeletal pain in SLE patients. A typical description is, "Joint involvement in SLE is similar to that of rheumatoid arthritis, primarily affecting the small joint of the hands, wrists and knees ... patients' symptoms (pain and stiffness) are usually out of proportion to the degree of synovitis present on physical examination" [17]. An inconsistency of symptoms and objective findings is always suggestive of central sensitization, as exemplified by FM. While FM is a common accompaniment of SLE [18], the SLE patients in this study were specifically screened not to have concomitant FM. The success of this screening was validated by the relatively low FIQR/SIQR scores compared to FM (29.6 in SLE vs. 56.6 in FM). The only SIQR question that significantly differentiated RA from SLE was sensitivity to "loud noises, bright lights, odors and cold." This may be a reflection of sensitivity to sunlight in SLE patients, but this cannot be inferred from this data set. The only pain location that significantly differentiated RA from SLE was neck pain, with 55% prevalence in SLE patients vs. 29% in RA patients. Other notable nonsignificant differences were a higher prevalence of foot pain (63% vs. 46%) and knee pain (53% vs. 39%) in SLE compared to RA. These differences may be due to the relatively small number of RA and SLE patients, but if confirmed in a larger data set, these differences could point to differences in the musculoskeletal symptoms of SLE and RA that have hitherto been opaque.

## Conclusions

This study analyzed data derived from patients with FM, SLE or RA who had completed the FIQR and/or SIQR and identified sites of pain in 24 locations. A combination of two SIQR questions ("tenderness to touch" and "difficulty sitting for 45 minutes") plus pain in four locations (lower back, neck, hands and arms) identified the correct diagnosis in 97% of patients. Overall, this report provides some pointers for distinguishing FM patients from patients with RA or SLE in clinical questionnaires and raises some potentially novel issues regarding musculoskeletal symptoms in SLE patients.

## Abbreviations

ACR: American College of Rheumatology; ANOVA: analysis of variance; DAS: Disease Activity Score; FIQ: Fibromyalgia Impact Questionnaire; FIQR: Revised Fibromyalgia Impact Questionnaire; FM: fibromyalgia; MDD: major depressive disorder; RA: rheumatoid arthritis; SIQR: Symptom Impact Questionnaire; SLE: systemic lupus erythematosus.

## Competing interests

The authors declare that they have no competing interests.

## Authors' contributions

RF and RB contributed equally to the design of the study, the analysis of the data and the writing of the manuscript.

## References

[B1] White KP Harth M Speechley M Ostbye T Testing an instrument to screen for fibromyalgia syndrome in general population studies: the London Fibromyalgia Epidemiology Study Screening Questionnaire J Rheumatol 1999 26 880 884 10229410

[B2] Häuser W Akritidou I Felde E Klauenberg S Maier C Hoffmann A Köllner V Hinz A [Steps towards a symptom-based diagnosis of fibromyalgia syndrome: symptom profiles of patients from different clinical settings] [in German] Z Rheumatol 2008 67 511 515 10.1007/s00393-008-0327-0 18830659

[B3] Perrot S Bouhassira D Fermanian J Cercle d'Etude de la Douleur en Rhumatologie Development and validation of the Fibromyalgia Rapid Screening Tool (FiRST) Pain 2010 150 250 256 10.1016/j.pain.2010.03.034 20488620

[B4] Wolfe F Clauw DJ Fitzcharles MA Goldenberg DL Katz RS Mease P Russell AS Russell IJ Winfield JB Yunus MB The American College of Rheumatology preliminary diagnostic criteria for fibromyalgia and measurement of symptom severity Arthritis Care Res (Hoboken) 2010 62 600 610 10.1002/acr.20140 20461783

[B5] Katz RS Wolfe F Michaud K Fibromyalgia diagnosis: a comparison of clinical, survey, and American College of Rheumatology criteria Arthritis Rheum 2006 54 169 176 10.1002/art.21533 16385512

[B6] Bennett RM Friend R Jones KD Ward R Han BK Ross RL The Revised Fibromyalgia Impact Questionnaire (FIQR): validation and psychometric properties Arthritis ResTher 2009 11 R120 10.1186/ar2783 PMC2745803 19664287

[B7] Burckhardt CS Clark SR Bennett RM The Fibromyalgia Impact Questionnaire: development and validation J Rheumatol 1991 18 728 733 1865419

[B8] Tabachnick BG Fidell LS Using Multivariate Statistics 1989 2 New York: Harper & Row

[B9] Choy EH Arnold LM Clauw DJ Crofford LJ Glass JM Simon LS Martin SA Strand CV Williams DA Mease PJ Content and criterion validity of the preliminary core dataset for clinical trials in fibromyalgia syndrome J Rheumatol 2009 36 2330 2334 10.3899/jrheum.090368 19820222 PMC3412585

[B10] Watson NF Buchwald D Goldberg J Noonan C Ellenbogen RG Neurologic signs and symptoms in fibromyalgia Arthritis Rheum 2009 60 2839 2844 10.1002/art.24772 19714636 PMC2769083

[B11] Wolfe F Smythe HA Yunus MB Bennett RM Bombardier C Goldenberg DL Tugwell P Campbell SM Abeles M Clark P Fam AG Farber SJ Fiechtner JJ Franklin CM Gatter RA Hamaty D Lessard J Lichtbroun AS Masi AT McCain GA Reynolds WJ Romano TJ Russell IJ Sheon RP The American College of Rheumatology 1990 Criteria for the Classification of Fibromyalgia: Report of the Multicenter Criteria Committee Arthritis Rheum 1990 33 160 172 10.1002/art.1780330203 2306288

[B12] Vrijhoef HJ Diederiks JP Spreeuwenberg C van der Linden S Applying low disease activity criteria using the DAS28 to assess stability in patients with rheumatoid arthritis Ann Rheum Dis 2003 62 419 422 10.1136/ard.62.5.419 12695152 PMC1754526

[B13] Felson DT Anderson JJ Boers M Bombardier C Chernoff M Fried B Furst D Goldsmith C Kieszak S Lightfoot R Paulus H Tugwell P Weinblatt M Widmark R Williams HJ Wolfe F The American College of Rheumatology preliminary core set of disease activity measures for rheumatoid arthritis clinical trials: The Committee on Outcome Measures in Rheumatoid Arthritis Clinical Trials Arthritis Rheum 1993 36 729 740 10.1002/art.1780360601 8507213

[B14] Tan EM Cohen AS Fries JF Masi AT McShane DJ Rothfield NF Schaller JG Talal N Winchester RJ The 1982 revised criteria for the classification of systemic lupus erythematosus Arthritis Rheum 1982 25 1271 1277 10.1002/art.1780251101 7138600

[B15] Pollard LC Kingsley GH Choy EH Scott DL Fibromyalgic rheumatoid arthritis and disease assessment Rheumatology (Oxford) 2010 49 924 928 10.1093/rheumatology/kep458 20100795

[B16] Kalichman L Hernández-Molina G Hand osteoarthritis: an epidemiological perspective Semin Arthritis Rheum 2010 39 465 476 10.1016/j.semarthrit.2009.03.001 19482338

[B17] Tassiulas IO Boumpas DT Firestein GS, Budd RC, Harris ED, McInnes IB, Ruddy S, Sergent JS Clinical features and treatment of systemic lupus erythematosus Kelley's Textbook of Rheumatology 2009 II 8 Philadelphia: Saunders/Elsevier 126921517306

[B18] Middleton GD McFarlin JE Lipsky PE The prevalence and clinical impact of fibromyalgia in systemic lupus erythematosus Arthritis Rheum 1994 37 1181 1188 10.1002/art.1780370812 8053957

